# Supplementary material for: Genome sequence and silkomics of the spindle ermine moth, Yponomeuta cagnagella, representing the early diverging lineage of the ditrysian Lepidoptera
Source: Commun Biol. 2022 Nov 23;5:1281. doi: 10.1038/s42003-022-04240-9 (PMC9684489; doi:10.1038/s42003-022-04240-9)
Supplement: Supplementary file 2 — Supplemental Material [file 42003_2022_4240_MOESM2_ESM.pdf]

Genome sequence and silkomics of the spindle ermine moth, *Yponomeuta cagnagella,* representing the early-diverging lineage of the ditrysian Lepidoptera

Anna Volenikova, Petr Nguyen, Peter Davey, Hana Sehadova, Barbara Kludkiewicz, Petr Koutecky, James R. Walters, Peter Roessingh, Irena Provaznikova, Michal Sery, Martina Zurovcova, Miluse Hradilova, Lenka Rouhova, Michal Zurovec

Supplementary information

Figure S1 – *Y. cagnagella* - larvae in protective nests, fully grown larva, cocoon and adult
Figure S2 – Example of flow cytometric histogram of propidium iodide fluorescence
Figure S3 – Estimate of heterozygosity and genome size using GenomeScope k-mer analysis
Figure S4 – Schematic drawings of the exon-intron structures of major *Y. cagnagella* silk genes
Figure S5 – Alignment of H-fibroin repeats
Figure S6 – Alignment of sericin 1 C-termini
Figure S7 –Alignment of H-fibroin N-termini
Figure S8 – Alignment of H-fibroin C-termini
Figure S9 – Alignment of L-fibroins
Figure S10 – Alignment of fibrohexamerins
Figure S11 – Alignment of mucin 1 C-termini
Figure S12 – Unedited northern blot images (for Figure 3).
Table S1 – List of primers
Table S2 – Analysis of contamination (Kraken2 software)
Table S3 – Major satellites identified in *Y. cagnagella* genome by the TAREAN pipeline.
Table S4 – RepeatMasker analysis of repeats
Table S5 – Estimates of evolutionary divergence
Table S6 – Data for Figure 4 (qPCR)

Figure S1 – *Yponomeuta cagnagella* - larvae in protective nests (A), fully grown larva (B), cocoon (C) and adult (D).

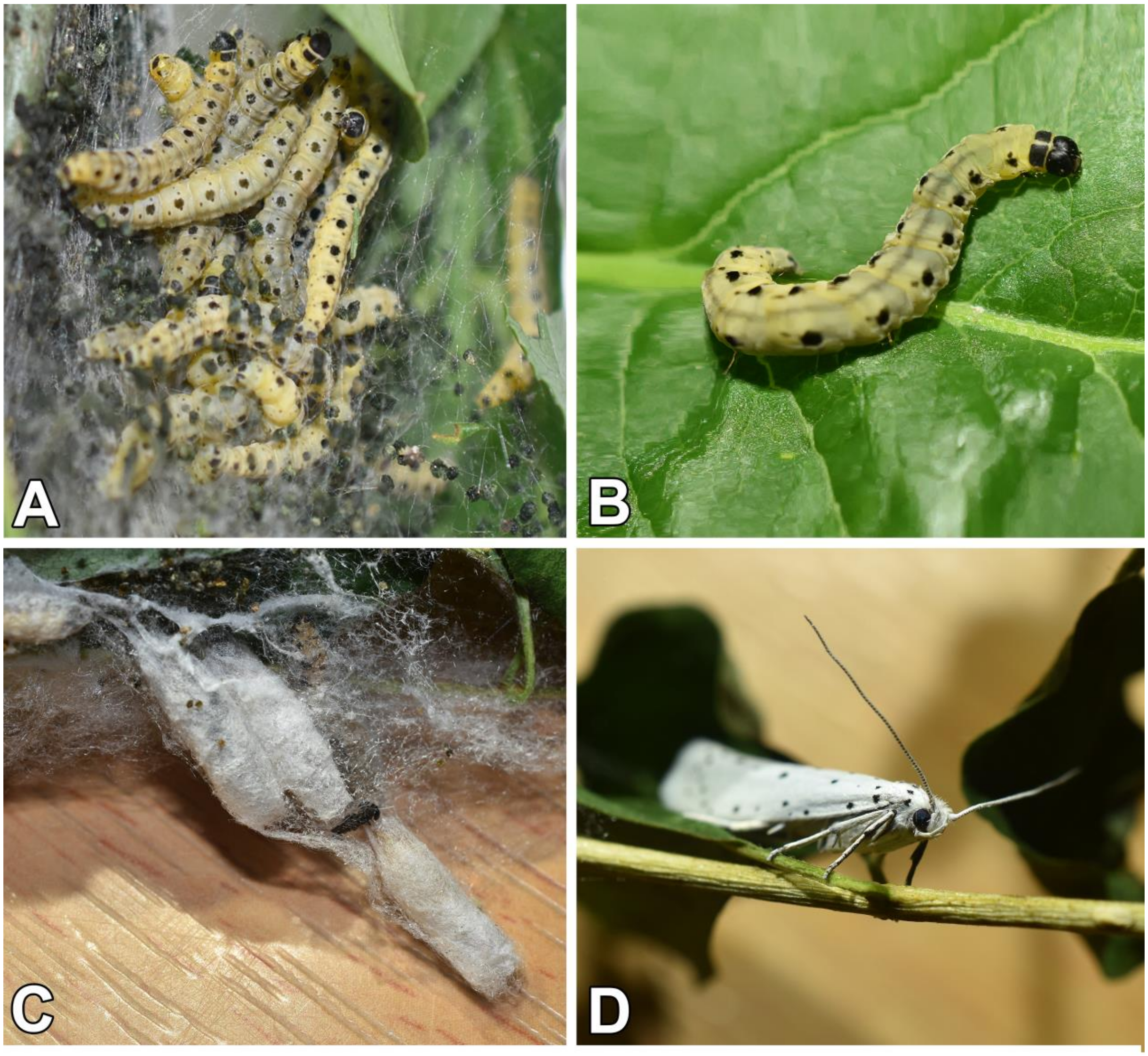

Figure S2. Estimation of *Y. cagnagella* genome size from nuclei isolated from brain tissue of *Y. cagnagella* and *E. kuehniella* (the internal standard). (**A**) Results show histograms of propidium iodide fluorescence. (**B**) Fluorescence vs SSC plot with polygonal gate used to sort out debris before histogram analysis. PI = propidium iodide, SSC = side scatter. 2C and weaker 4C peaks of both species are clearly visible.

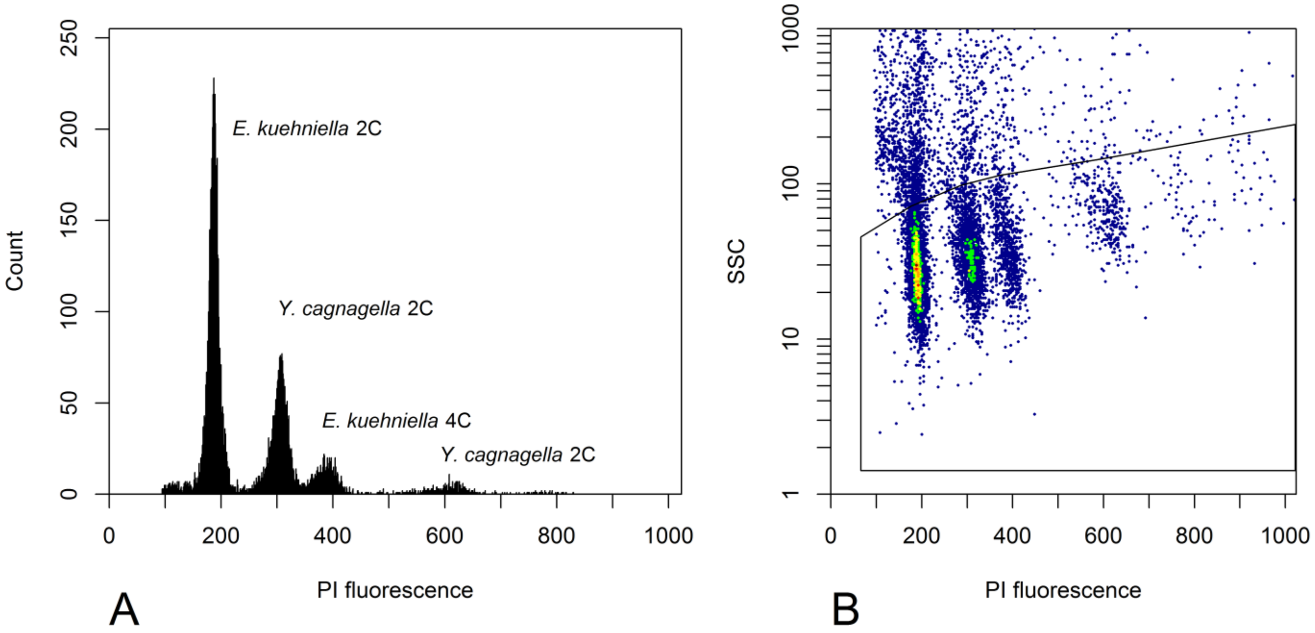

Figure S3 – K-mer survey of *Yponomeuta cagnagella* genome. GenomeScope analysis with k-mer of length 31 revealed high genome heterozygosity (1.29%) with an estimated haploid genome size of 508.5 Mbp.

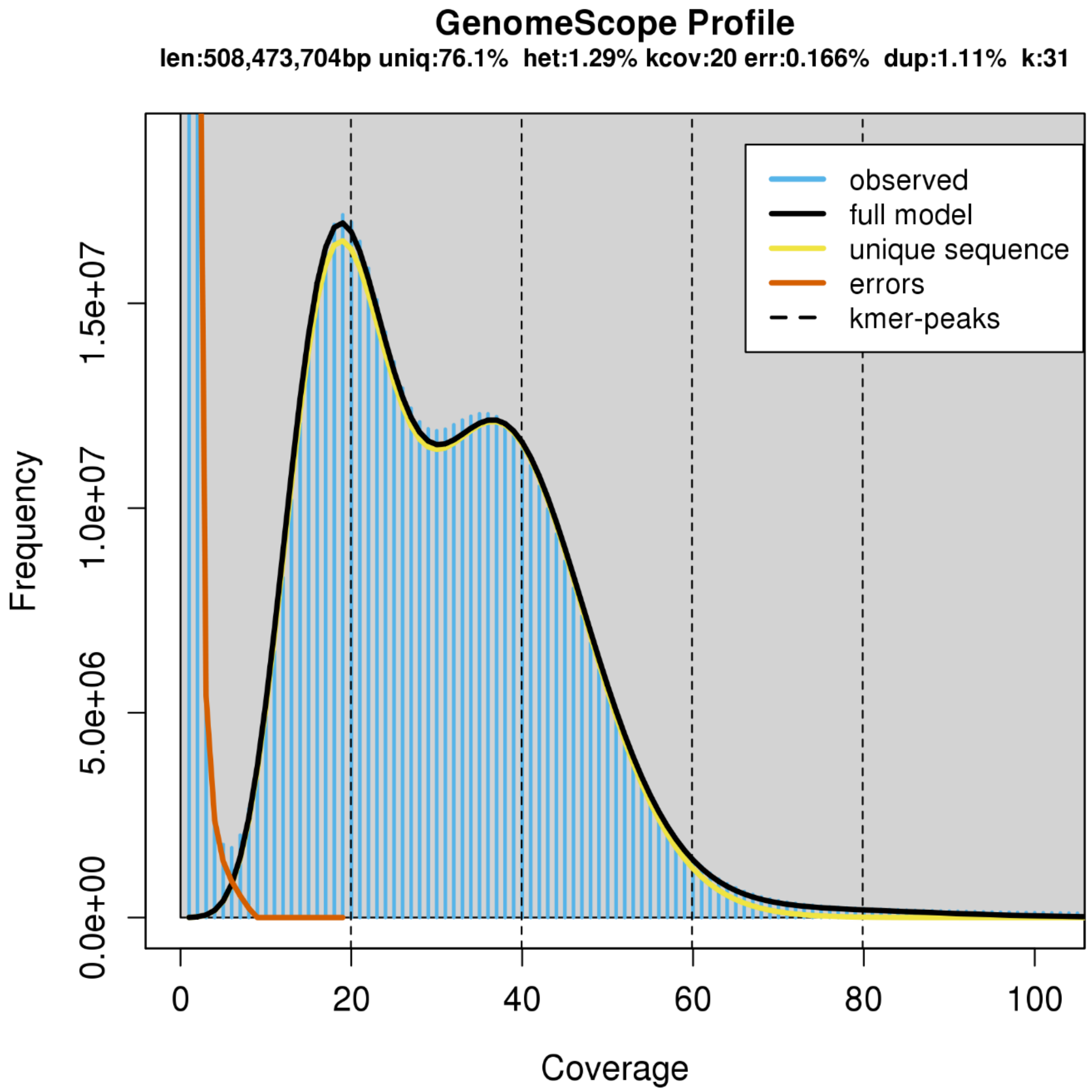

Figure S4. Schematic representations of the exon-intron structures of the major silk genes of *Y. cagnagella*. The full names of each gene are listed in Table 2. The 5' ends of the genes are on the left. The scale in kilobases is given below the figure.

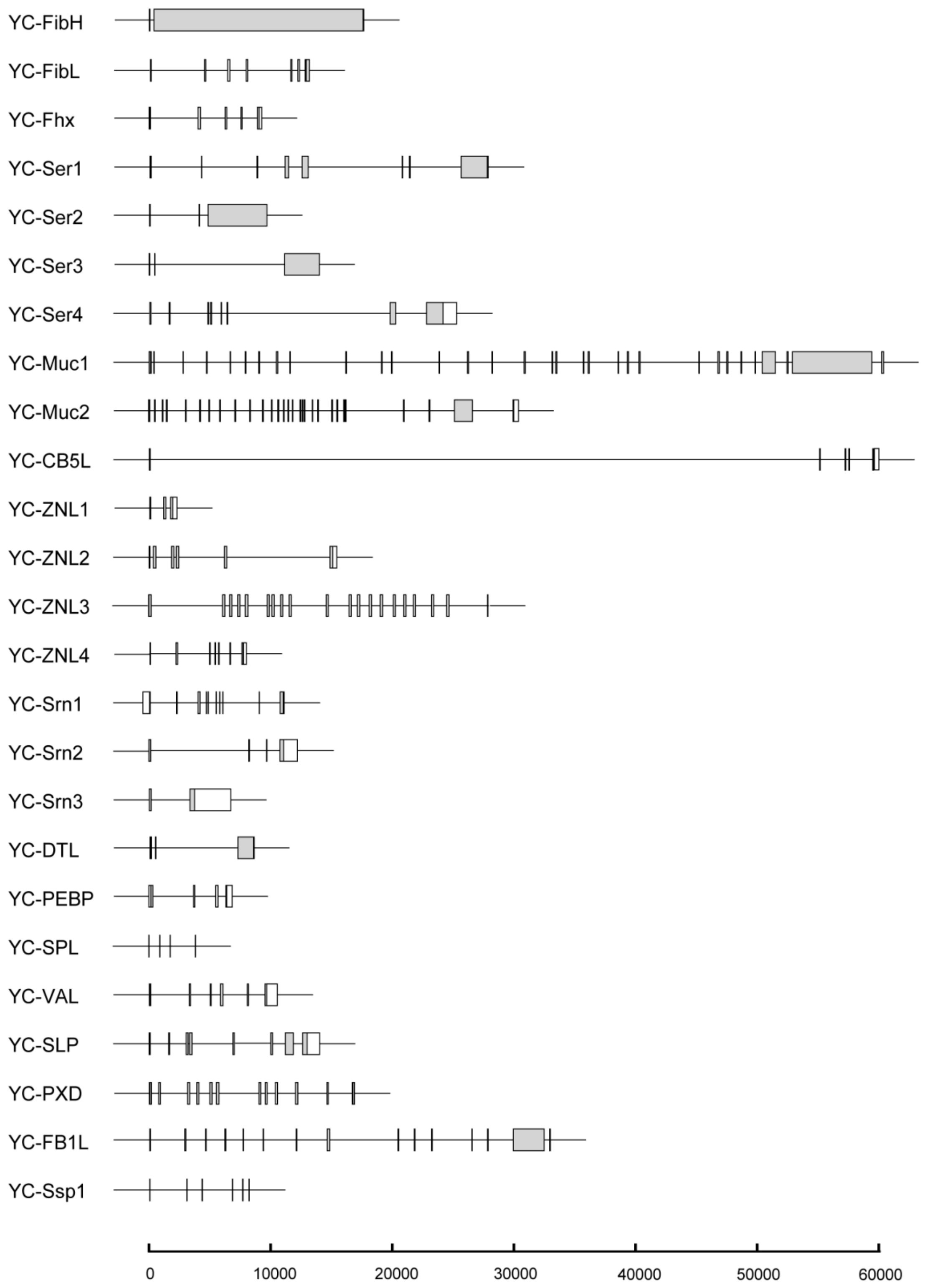

Figure S5. Multiple alignment of central repeated sequences of H-fibroin proteins. Genbank accession numbers are as follows: *Bombyx mori* M_001113262, *Pseudoips (Bena) prasinana* MW373748, *Tineola bisselliella* MW244680, *Eumeta japonica* LC507818, *Galleria mellonella* AH009792, *Yponomeuta cagnagella* MZ959145, *T. bisselliella* MW244680, *A. yamamai* AB542805. The residues that are identical or similar in most of these sequences are highlighted. The types of fibroin structural classes are marked on the left.

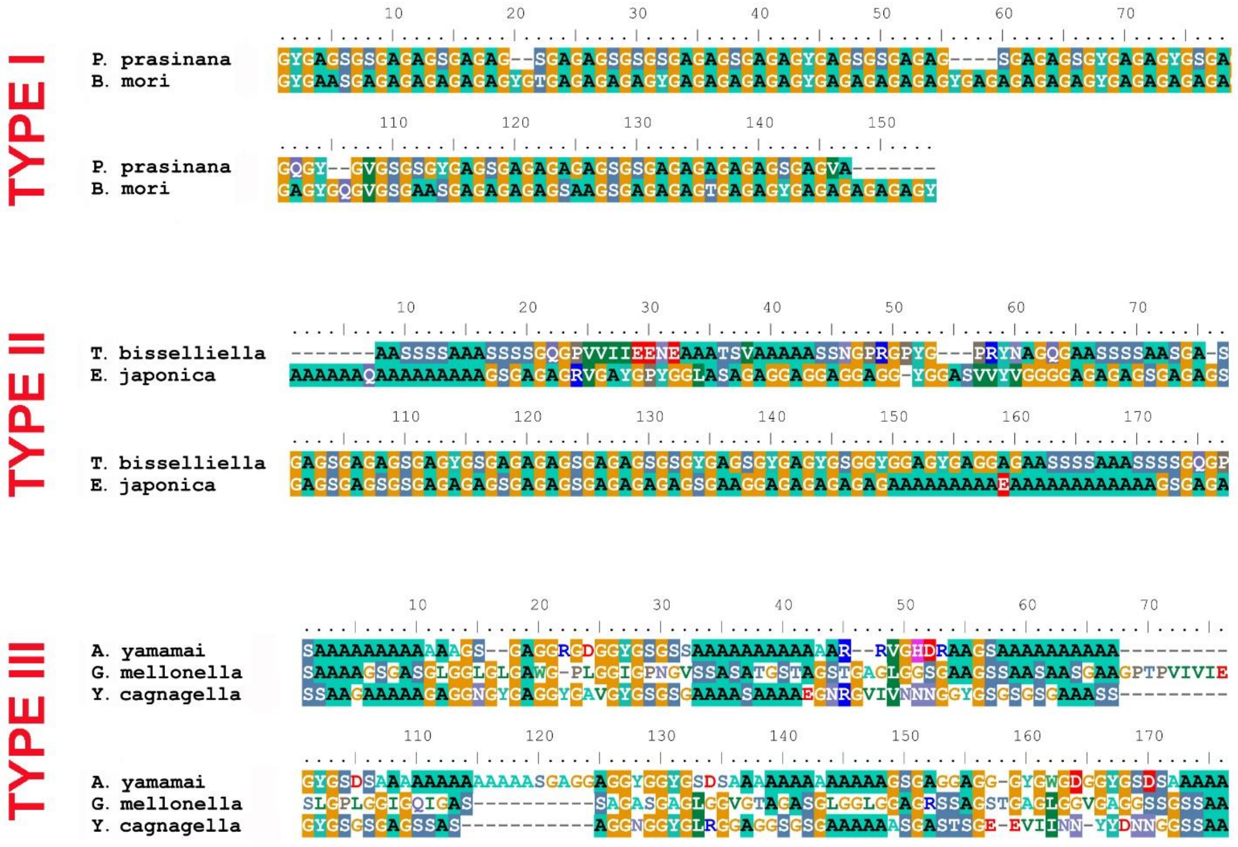

Figure S6. Multiple alignment of C-terminal sequences of sericin-1-like proteins. Genbank accession numbers are as follows: *Antheraea yamamai* LC085887, *Bombyx mori* XM_038013631, *Pseudoips prasinana A* MW373764, *Pseudoips prasinana B* MW373765, *Galleria mellonella A* MG770315, *Galleria mellonella B* MG770316, *Yponomeuta cagnagella* MZ927540, *Tineola bisselliella* MW244705. Residues that are identical or similar in most of these sequences are shaded in color. The asterisks indicate conserved cysteine residues.

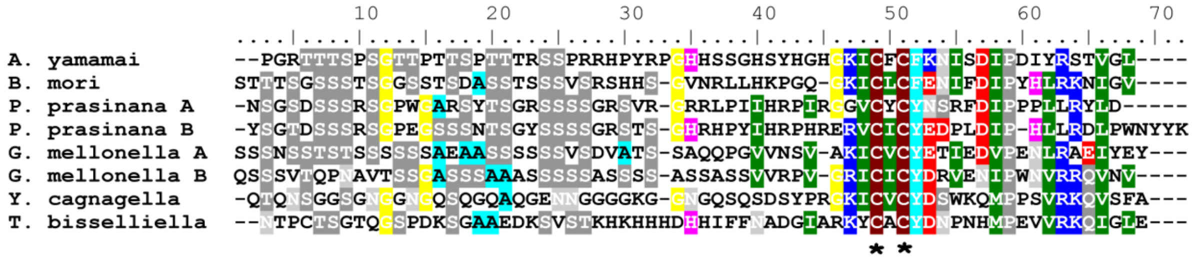

Figure S7. Alignment of the N-terminal sequences of H-fibroin (moths and caddisflies). Genbank accession numbers are as follows: *Bombyx mori* M_001113262, *Pseudoips (Bena) prasinana* MW373748, *Galleria mellonella* AH009792, *Yponomeuta cagnagella* MZ959145, *Tineola bisselliella* MW244680, *Phymatopus (Hepialus) californicus* GU144520, *Plectrocnemia conspersa* OL589410 and *Hydropsyche angustipennis* AB354592. The residues that are identical or similar in most of these sequences are highlighted.

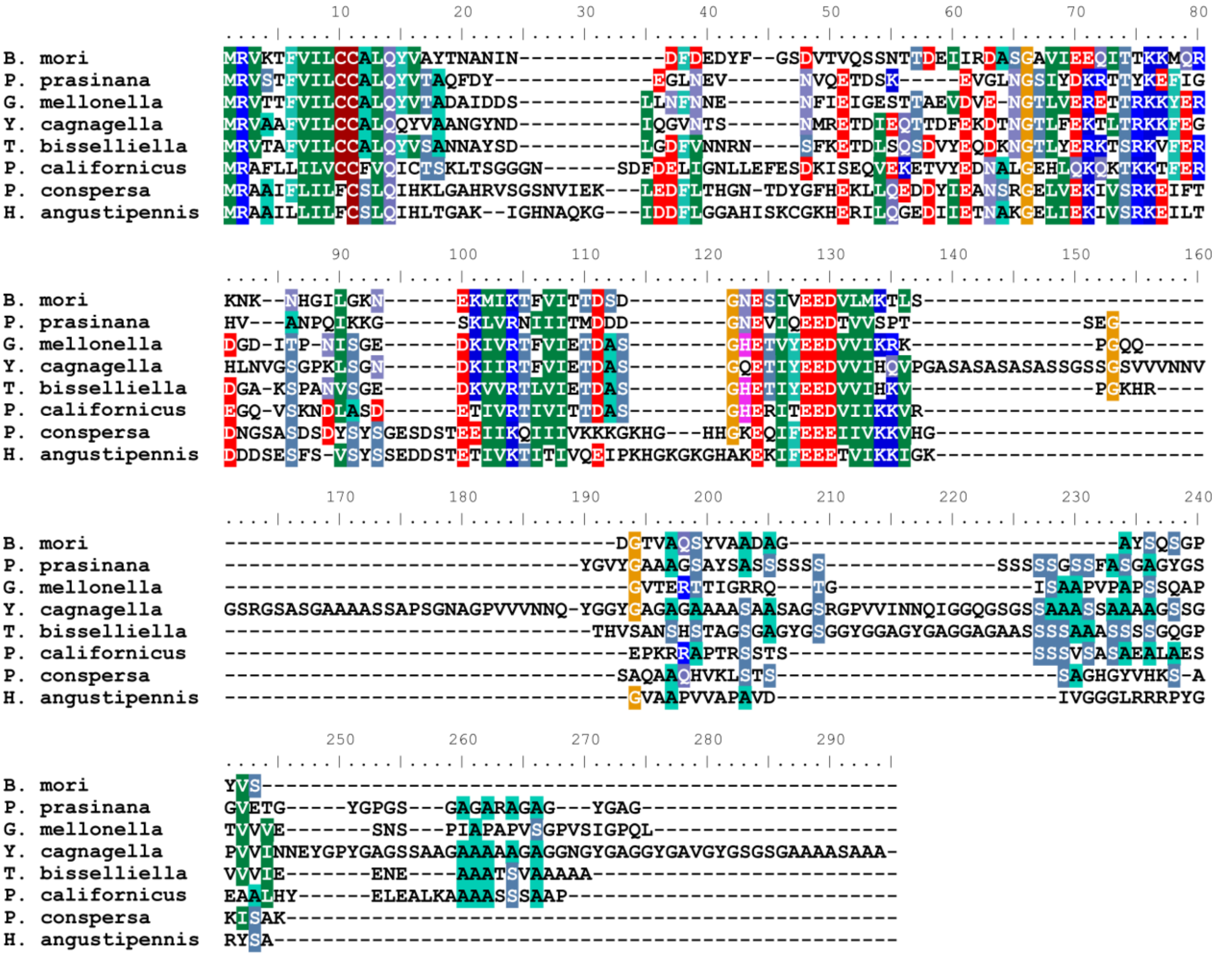

Figure S8. Alignment of the C-terminal sequences of H-fibroin (moths and caddisflies). The Genbank accession numbers are as follows: *Bombyx mori* M_001113262, *Pseudoips (Bena) prasinana* MW373748, *Galleria mellonella* AH009792, *Yponomeuta cagnagella* MZ959145, *Tineola bisselliella* MW244680, *Phymatopus (Hepialus) californicus* GU144520, *Plectrocnemia conspersa* OL589410, *Hydropsyche angustipennis* AB354592. The residues that are identical in most of these sequences are shaded. Three conserved cysteine residues are indicated with an asterisk.

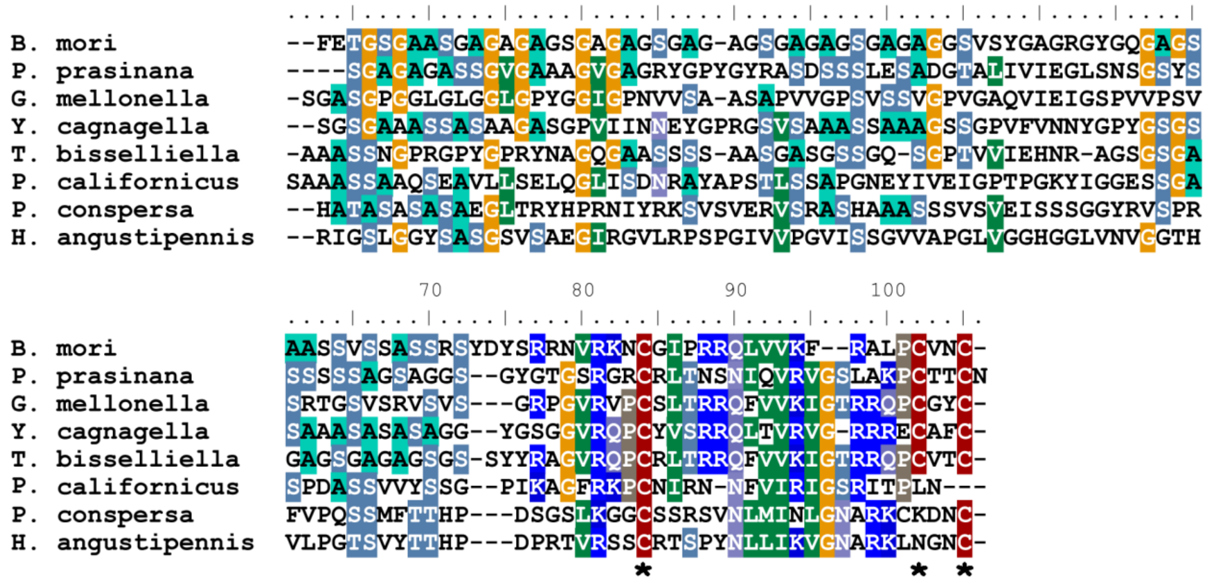

Figure S9. Multiple alignment of L-fibroin protein sequences (moths and caddisflies). Genbank accession numbers are as follows: *Bombyx mori* NM_001044023, *Papilio xuthus* AB001824, *Galleria. mellonella* light-chain fibroin S77817, *Yponomeuta cagnagella* MZ959144, *Tineolla bisselliella* MW244681, *Phymatopus (Hepialus) californicus* GU180675, *Plectrocnemia conspersa* OL589402. The residues that are identical or similar in most of these sequences are highlighted in color.

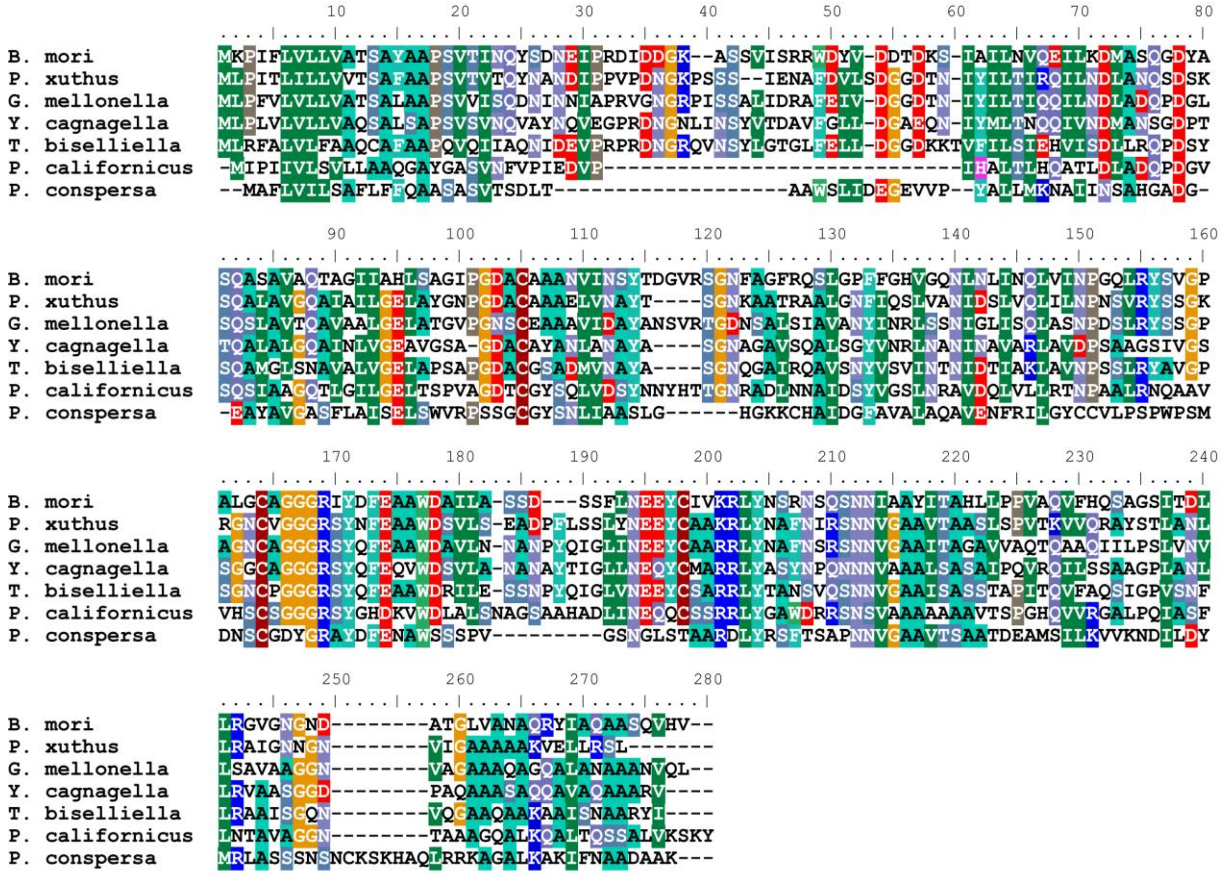

Figure S10. Multiple alignment of fibrohexamerin (P25) protein sequences. Genbank accession numbers are as follows: *Bombyx mori* NM_ NM_001145941, *Papilio xuthus* AB001825, *G. mellonella* fibrohexamerin (P25) AF009827, *Yponomeuta cagnagella* MZ959146, and *Tineola bisselliella* MW244682. The residues, which are identical in most of these sequences, are shaded in color.

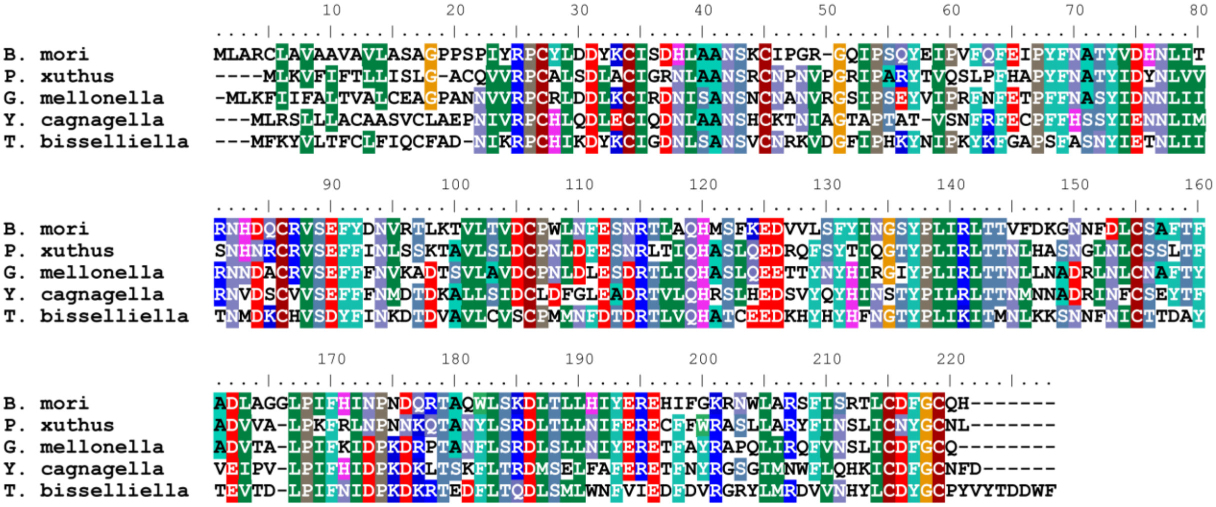

Figure S11. Alignment of the C-terminal sequences of mucin-1-like proteins. The Genbank accession numbers are as follows: *Bombyx mori* XM_038013902, *Manduca sexta* titin (LOC115444707. *Galleria mellonella A* mucin-like MG770312.1, *Galleria mellonella B* GPI-anchored adhesin-like protein XM_026908157, *Yponomeuta cagnagella* MZ981775, *Tineola bisselliella* mucin 1 MW244683.1 Residues identical in the majority of these sequences are highlighted. The asterisks indicate conserved cysteine residues.

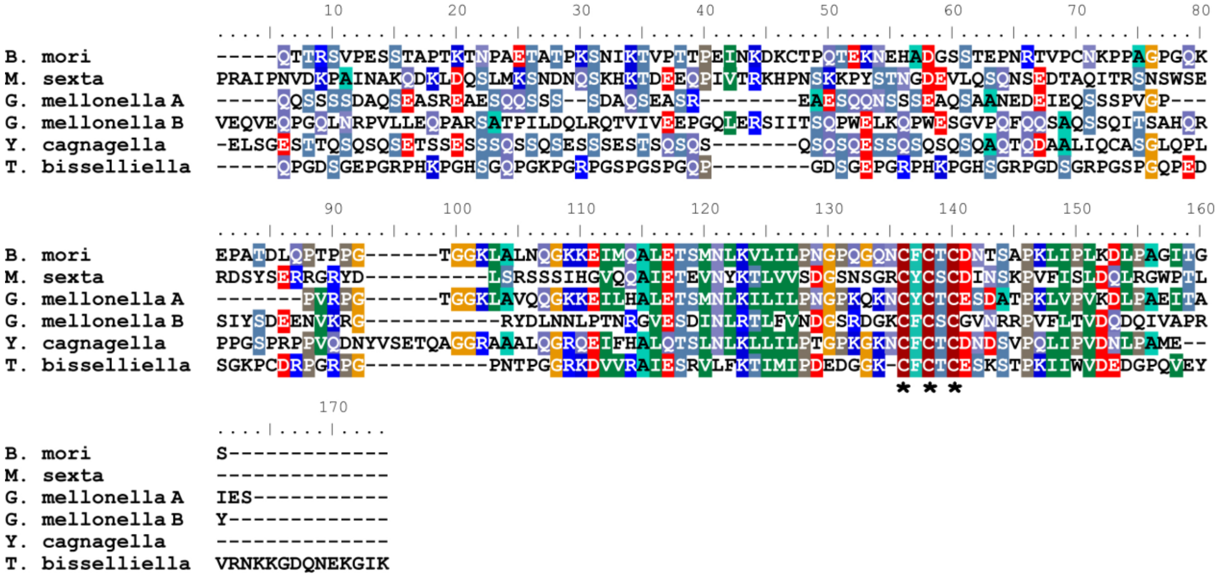

Figure S12. Unedited northern blot images (for Figure 3). Symbols greater (>) or less (<) than mark the position of 1.9/2.0 kb band.

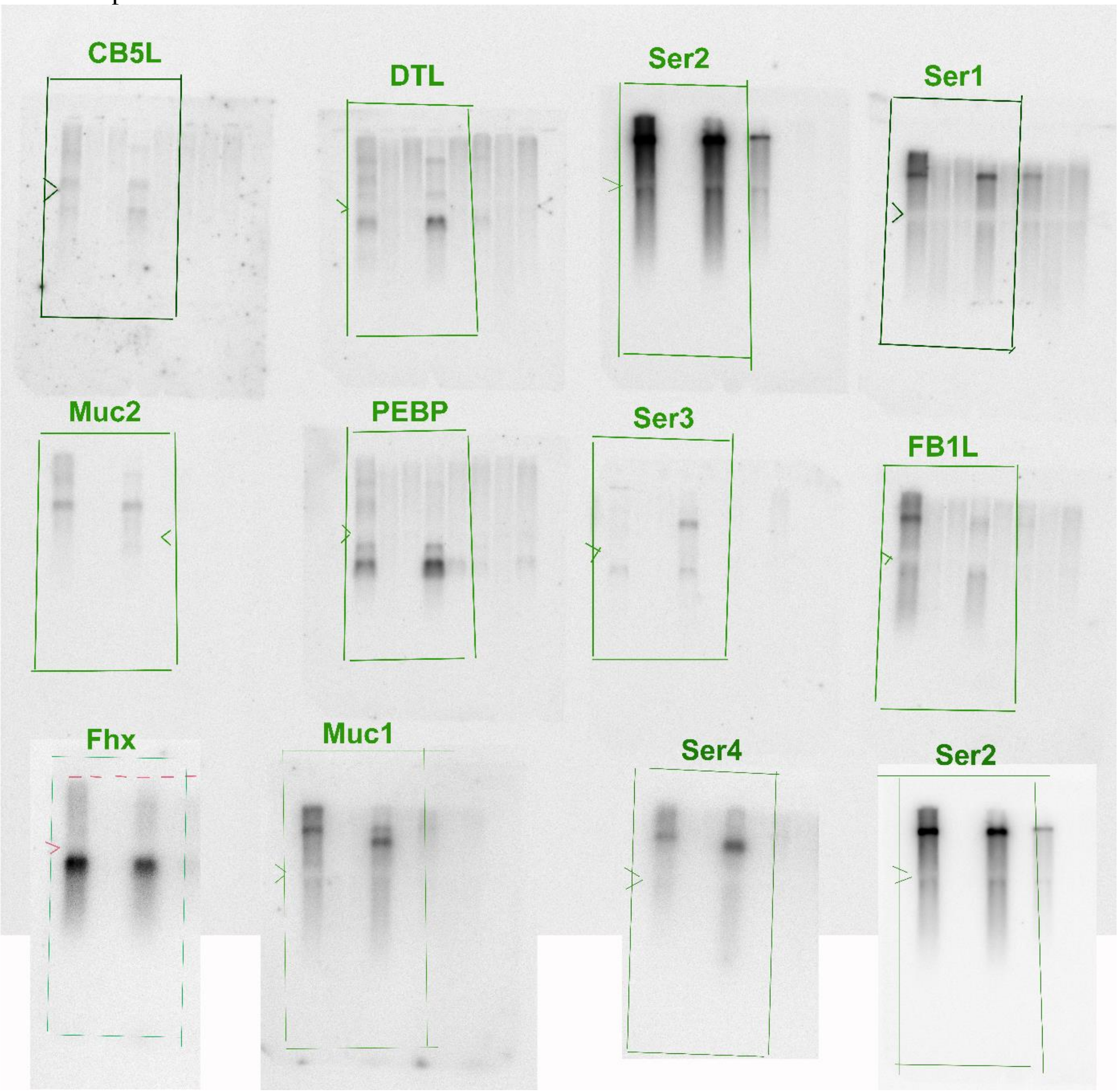

Table S1: List of primers used for cDNA probes (for northern blotting) and for qPCR

**NORTHERN BLOTS**			
Sericin 1 (YC-Ser1)	Fw	**CAATGGACAAAGCCAAGGCCCA**	65°C
	Rv	**GAGACTTGTTTTCTAACGCTTGGC**	65°C
Sericin 2 (YC-Ser2)	Fw	**GATGGCAGCGAGGAGGTGGAC**	67°C
	Rv	**CCTGCGATTGGGCCGAAGAAAC**	67°C
Sericin 3 ((YC-Ser3)	Fw	**ATTGAACGCGAAAATGCTGTAAGG**	65°C
	Rv	**TTTGCGTTGTTCGTAGTGGTGTC**	65°C
Mucin 1 (YC-Muc1)	Fw	**GAGCATCGCCGTCGTCAAGAAG**	67°C
	Rv	**GGTCGCGGCGTGTCGGATGTT**	65°C
Mucin2 (YC-Muc2)	Fw	**TGGGCCAGAATTTGAACCGAGATA**	67°C
	Rv	**CCACTTCCGTTCACACCTTTACC**	67°C
CytB-like (YC-CB5L)	Fw	**GGGCTGCGAGGACACGGTTATC**	69°C
	Rv	**TCAATCCCCTTCTTCATTTGCTACA**	67°C
PEBP-binding p (YC-PEBP)	Fw	**GTTGAAGACGCGTTCAGGAGA**	61°C
	Rv	**AAAAAGCCGCTTATTGGACTTTG**	61°C
Fibrillin1-like (YC-FB1L)	Fw	**TGGCGGCGGAGGAGGATGG**	63°C
	Rv	**CGTTTCTTAGCCGACTTGAGGT**	63°C
Fibrohexamerin (YC-Fhx)	Fw	**AAGCCTACTTTTGGCTTGTGCT**	61°C
	Rv	**AGCGATCGATGCTGGAGGAC**	61°C
Sericin 4 (YC-Ser4)	Fw	**ACCGGGAGCGTCGTCAAGGAG**	67°C
	Rv	**TTTGAACCGCTAGAAGAAGAGTCA**	65°C
Dentin-like (YC-DTL)	Fw	**ATTTCTATTATCGCGGGCACCTT**	63°C
	Rv	**TTTCTCGCTACTTGTTTCTTCTGA**	63°C
**Q-PCR**			
Antichymotrypsin-1-like (ACHP)	Fw	**GTCCTCGGCGTATATTTCCTTC**	53,7 °C
	Rv	**TGCTCCGCCCTCATTCC**	54 °C
Sulfatase B (YC-SLP)	Fw	**AGCTCCTGCCGTCCTACCTT**	54,5 °C
	Rv	**GGCCCCATCGAGAAATCC**	53,9 °C
Zonadhesin-like 1 (YC-ZNL1)	Fw	**GCCCTATTGCCCGACTGAC**	54,3 °C
	Rv	**GCCTTCCCTGCATTGACATT**	53,9 °C
Trypsin-like proteinase (YC-TRPL)	Fw	**TTACCGCCCGGACTGCTA**	54 °C
	Rv	**CGGGGCGGATACCTACTGT**	53,8 °C
Venom allergen 5-like (YC-VAL)	Fw	**ACGGCCGGTGGAACAGA**	54,3 °C
	Rv	**GGTCGCCGTAGGGATGG**	53,3 °C
Fibrillin1-like (YC-FB1L)	Fw	**GCGCTGGTTTTCTTCTTGAC**	51,7 °C
	Rv	**CGCCGCGACAGTAACAGT**	52 °C
Zonadhesin-like 2 (YC-ZNL2)	Fw	**TCCGGTGACACATCGCTAAA**	53,9 °C
	Rv	**GGACCGGCTATAAAAACAAGAAC**	53,1 °C
Zonadhesin-like 3 (YC-ZNL3)	Fw	**CCGACGGCTCCTGTATCAA**	53,4 °C
	Rv	**GTGCATGCCCGGCTATTATC**	54,5 °C

Fibrillin2-like (YC-FB2L)	Fw	**GGCTGCGGCTTAGGAATC**	52,3 °C
	Rv	**ATCAGCGGCACCAAACAA**	52,1 °C
Bangles and beads (YC-BNB)	Fw	**CCGCGAAGGCCATACATAATA**	54,4 °C
	Rv	**GTGGCAGCTCGCAACTTTTT**	54,6 °C
Zonadhesin-like 4 (YC-ZNL4)	Fw	**AGCCGTTGACTGCGTTAGG**	52,8 °C
	Rv	**CTCCGGGCTGCGTCTGT**	54,2 °C
Serine protease (YC-SPL)	Fw	**GGGCAGGCCACATACCAGT**	54,5 °C
	Rv	**TGCGGCCAGGAGACCAC**	54,9 °C
Imaginal Disc Growth Factor-like (YC-Idgf)	Fw	**TCCGGAGTGCAGCCAGTAGT**	54,6 °C
	Rv	**CCGGAGCGAAACATCAAG**	51,3 °C
Fibroin L (YC-FibL)	Fw	**ACGGCGGGCCATACAGT**	53,4 °C
	Rv	**GCGGCGGCAGGTCATAC**	54,8 °C
Agrin-like (YC-AGRL)	Fw	**GTTGCCCCGCCATCTTG**	54,4 °C
	Rv	**ACCGCATCCGCCGTAGTA**	53,8 °C
Peroxidase (YC-PXD)	Fw	**CAGCACGGGTTTATTCTCTTCA**	53,7 °C
	Rv	**GATTTGGCTCTCCGACAGTTG**	53,8 °C
Elongation factor 1 (YC-EF1)	Fw	**CTGGAGGGCTTCGTGGTG**	53,6 °C
	Rv	**TGGGCCGTGTGGAGACTG**	54,8 °C

Table S2: Kraken2 contamination analysis. List of contigs assigned outside Lepidoptera and their classification, length and k-mer composition.

CONTIG #	SPECIES	LENGTH (bp)	K-MER ASSIGNMENT (taxon ID*: number of k-mers)
contig_15361	Homo sapiens (taxid 9606)	1857	0:574 9606:3 0:512 9606:5 0:729
contig_28074	Streptococcus gwangjuense (taxid 1433513)	5029	0:3027 9606:3 0:217 1433513:5 0:1743
contig_28898	Wigglesworthia glossinidia endosymbiont (taxid 1142511)	13091	0:4989 1142511:5 0:40 7088:3 0:4494 7088:1 0:3525
contig_8686	Paenibacillus sp. JZ16 (taxid 1906272)	4150	0:436 1906272:5 0:2048 7088:1 0:5 7088:2 0:1619
contig_31422	Bacteria (taxid 2)	3656	0:1319 1685010:5 0:366 7088:4 0:168 562:5 0:1755
contig_39886	Actinomycetia (taxid 1760)	12598	0:736 1297793:1 0:5624 7088:1 0:1342 2591108:5 0:392 31958:5 0:4458
contig_12515	Polynucleobacter sp. MG-9-Vaara-E2 (taxid 2770235)	1476	0:972 2770235:3 0:387 7088:1 0:79
contig_2341	Streptomyces sp. RTd22 (taxid 1841249)	13555	1841249:5 0:1 1841249:5 0:1 1841249:5 0:1 1841249:3 0:34 1841249:5 0:1 1841249:5 0:1 1841249:5 0:1 1841249:5<br>0:1 1841249:5 0:1 1841249:5 0:1 1841249:5 0:1 1841249:5 0:1 1841249:5 0:1 1841249:5 0:1 1841249:5 0:1 1841249:5<br>0:854 7088:17 0:48 7088:1 0:362 7088:5 0:71 7088:27 0:43 7088:5 0:504 1283:5 0:1600 644282:3 0:203 39695:2
contig_20880	Homo sapiens (taxid 9606)	2673	0:91 7088:5 0:1074 9606:2 0:130 83451:2 0:69 9606:3 0:1237 9606:2 0:24
contig_3208	Paracoccus jeotgali (taxid 2065379)	4529	0:407 7088:5 0:2079 7088:5 0:5 28211:4 398580:4 1224:5 28211:2 0:83 7088:2 0:47 1178515:1 2704463:5 2:1<br>2626047:5 0:368 1224:5 2:1 2065379:5 0:341 2686360:1 0:132 1224:10 0:30 7088:5 0:39 2709394:1 0:897
contig_18674	Homo sapiens (taxid 9606)	3541	0:377 9606:4 0:985 7088:1 0:2140
contig_22260	Streptococcus parasuis (taxid 1501662)	4456	0:451 1501662:5 0:958 7088:1 0:599 69965:3 0:1264 7088:1 0:1140
contig_15459	Candidatus Izimaplasma sp. HR1 (taxid 1541959)	4550	0:587 324057:1 0:1047 1541959:3 0:1234 7088:1 0:1643
contig_29402	Nocardioides anomalus (taxid 2712223)	1341	0:399 2675773:2 0:260 7088:4 2712223:5 0:637
contig_6903	Homo sapiens (taxid 9606)	9469	0:819 7088:6 0:1 7088:5 0:116 7088:8 9606:92 7088:14 0:15 7088:5 9606:16 7088:10 0:21 7088:5 0:16 131567:5 0:54<br>7088:9 131567:5 0:38 7088:5 9606:14 7088:5 131567:5 0:16 7088:2 0:45 7088:5 131567:4 0:38 7088:5 9606:45 0:5<br>9606:5 0:19 131567:5 0:5 7088:1 0:31 131567:5 0:21 7088:5 0:5 7088:5 0:15 7088:3 9606:14 7088:2 131567:5 0:26
contig_19034	Homo sapiens (taxid 9606)	3000	0:471 9606:1 0:750 9606:4 0:453 7088:1 0:34 7088:3 0:1249
contig_14207	Homo sapiens (taxid 9606)	3472	0:553 7088:2 9606:4 0:71 2732590:5 0:1504 9606:7 0:93 9606:2 0:1197
contig_32405	Pseudoalteromonas sp. DL-6 (taxid 1390185)	4635	0:767 7088:3 0:2380 1390185:5 0:1446
contig_31368	Pasteurella (taxid 745)	756	0:258 7088:4 0:205 745:5 0:250
contig_24653	Neptunomonas concharum (taxid 1031538)	5200	0:2397 2662362:2 0:87 157692:2 0:791 272774:2 0:190 1031538:7 0:414 7088:2 0:609 197:5 0:658
contig_16078	Homo sapiens (taxid 9606)	5732	0:4204 9606:3 0:987 371142:2 0:502
contig_31359	unclassified Synechococcus (taxid 2626047)	5344	0:3216 2626047:2 0:1513 7088:1 0:578
contig_16937	Bacteria (taxid 2)	2894	0:1977 571430:5 0:560 185978:5 0:214 7088:1 0:98
contig_7635	Flavobacterium crocinum (taxid 2183896)	2351	0:37 2183896:3 0:1821 7088:1 0:62 7088:1 0:392
contig_9531	Homo sapiens (taxid 9606)	3367	0:221 9606:4 0:994 7088:2 0:2112
contig_20174	Campylobacter hepaticus (taxid 1813019)	637	0:309 1813019:5 0:282 7088:1 0:6
contig_24662	Aequorivita sp. H23M31 (taxid 2494375)	4729	2494375:1 2:5 2494375:1 2:5 2494375:1 2:5 2494375:1 2:5 2494375:1 2:5 2494375:1 2:5 2494375:1 2:5 2494375:1 2:5<br>2494375:1 2:5 2494375:1 0:986 7088:1 0:3 7088:10 0:2 7088:2 0:42
contig_32948	Gammaproteobacteria (taxid 1236)	11113	2:5 1236:1 2:5 1236:1 2:5 1236:1 2:5 1236:1 2:5 1236:1 2:5 1236:1 2:5 1236:1 2:5 1236:1 2:5 1236:1 2:5 1236:1 2:5<br>1236:1 2:5 1236:1 2:5 1236:1 2:5 1236:1 2:5 1236:1 2:5 1236:1 2:5 1236:1 2:5 1236:1 2:5 1236:1 2:5 1236:1 2:5 1236:1<br>2:5 1236:1 2:5 1236:1 2:5 1236:1 2:5 1236:1 2:5 1236:1 2:5 1236:1 2:5 1236:1 2:5 1236:1 2:5 1236:1 2:5 1236:1 2:5<br>1236:1 2:5 1236:1 2:5 1236:1 2:5 1236:1 2:5 1236:1 2:5 1236:1 2:5 1236:1 2:5 1236:1 2:5 1236:1 2:5 1236:1 2:5 1236:1<br>2:5 1236:1 2:5 1236:1 2:5 1236:1 2:5 1236:1 2:5 1236:1 2:5 1236:1 2:5 1236:1 2:5 1236:1 2:5 1236:1 2:5 1236:1 2:5<br>1236:1 2:5 1236:1 2:5 1236:1 2:5 1236:1 2:5 1236:1 2:5 1236:1 2:5 1236:1 2:5 1236:1 2:5 1236:1 2:5 1236:1 2:5 1236:1
contig_13357	Candidatus Blochmannia vafer str. BVAF (taxid 859654)	4945	0:450 1122611:3 0:859 1826607:1 0:2152 859654:4 0:1442
contig_6251	Homo sapiens (taxid 9606)	4688	0:606 9606:5 0:3394 816:1 0:648
contig_2866	Vibrio cidicii (taxid 1763883)	32741	7088:5 0:1233 7088:2 0:1 7088:5 0:1853 28197:3 0:821 7088:1 0:104 7088:1 0:1570 564:5 0:276 7088:5 0:405<br>2812896:3 0:99 7088:5 0:10 7088:5 0:2 7088:6 0:12 7088:5 0:1 7088:3 0:28 7088:7 0:17 7088:1 0:17 96561:1 0:5<br>1763883:3 0:1 1763883:5 0:1 1763883:5 0:1 1763883:5 0:1 1763883:5 0:1 1763883:5 0:1 1763883:5 0:1 1763883:5 0:1<br>1763883:5 0:1 1763883:5 0:1 1763883:5 0:1 1763883:5 0:1 1763883:5 0:1 1763883:5 0:1 1763883:5 0:1 1763883:5 0:1<br>1763883:5 0:1 1763883:5 0:1 1763883:4 0:73 1763883:5 0:1 1763883:5 0:1 1763883:5 0:1 1763883:5 0:1 1763883:5<br>0:1 1763883:5 0:1 1763883:5 0:1 1763883:5 0:1 1763883:5 0:1 1763883:5 0:1 1763883:5 0:1 1763883:5 0:1 1763883:5<br>0:1 1763883:5 0:1 1763883:5 0:1 1763883:5 0:1 1763883:5 0:1 1763883:5 0:1 1763883:5 0:1 1763883:5 0:1 1763883:5<br>0:1 1763883:5 0:1 1763883:5 0:1 1763883:5 0:1 1763883:5 0:1 1763883:5 0:1 1763883:5 0:1 1763883:5 0:1 1763883:5<br>0:1 1763883:5 0:1 1763883:5 0:1 1763883:5 0:1 1763883:5 0:1 1763883:5 0:1 1763883:5 0:1 1763883:7 0:6 7088:1<br>0:21 1763883:5 0:1 1763883:5 0:1 1763883:5 0:1 1763883:5 0:1 1763883:5 0:1 1763883:5 0:1 1763883:5 0:1<br>1763883:5 0:1 1763883:5 0:1 1763883:7 0:26 1763883:3 0:1 1763883:5 0:1 1763883:5 0:1 1763883:5 0:1 1763883:5<br>0:1 1763883:5 0:1 1763883:5 0:1 1763883:5 0:1 1763883:5 0:1 1763883:5 0:1 1763883:5 0:1 1763883:5 0:1 1763883:5<br>0:1 1763883:5 0:1 1763883:5 0:1 1763883:5 0:1 1763883:5 0:1 1763883:5 0:1 1763883:5 0:1 1763883:5 0:1 1763883:5<br>0:1 1763883:5 0:1 1763883:5 0:1 1763883:5 0:1 1763883:5 0:1 1763883:5 0:1 1763883:5 0:1 1763883:5 0:1 1763883:5<br>0:1 1763883:5 0:1 1763883:5 0:1 1763883:5 0:1 1763883:5 0:1 1763883:5 0:1 1763883:5 0:1 1763883:5 0:1 1763883:5<br>0:1 1763883:5 0:1 1763883:5 0:1 1763883:5 0:1 1763883:5 0:1 1763883:5 0:1 1763883:5 0:1 1763883:5 0:1 1763883:5<br>0:1 1763883:5 0:1 1763883:5 0:1 1763883:5 0:1 1763883:5 0:1 1763883:5 0:1 1763883:5 0:1 1763883:5 0:1 1763883:5<br>0:1 1763883:5 0:1 1763883:5 0:1 1763883:5 0:1 1763883:5 0:1 1763883:5 0:1 1763883:5 0:1 1763883:5 0:1 1763883:5<br>0:1 1763883:5 0:1 1763883:5 0:1 1763883:5 0:1 1763883:5 0:1 1763883:5 0:1 1763883:5 0:1 1763883:5 0:1 1763883:5<br>0:1 1763883:2 0:29 2:4 0:25 1763883:5 0:1 1763883:5 0:1 1763883:5 0:1 1763883:5 0:1 1763883:5 0:1 1763883:5 0:1<br>1763883:5 0:1 1763883:5 0:1 1763883:5 0:1 1763883:5 0:1 1763883:5 0:1 1763883:5 0:8 7088:1 0:23 1763883:3 0:1
contig_24729	Geobacillus (taxid 129337)	2302	0:1043 2799680:3 0:703 129337:5 0:514

***taxon ID 0 = unclassified; 7088 = Lepidoptera**

Table S3: Major satellites identified in *Yponomeuta cagnagella* genome by the TAREAN pipeline.

Satellite ID	Consensus length (bp)	Genome proportion (%)	Satellite probability	Accession number
**Ycag_sat_cl11**	179	0.40	0.928	
**Ycag_sat_cl24**	706	0.23	0.986	
**Ycag_sat_cl27**	780	0.20	0.029	
**Ycag_sat_cl40**	854	0.13	0.101	
**Ycag_sat_cl54**	607	0.09	0.211	
**Ycag_sat_cl98**	341	0.04	0.986	
**Ycag_sat_cl214**	256	0.01	0.660	

>Ycag_sat_cl11_consensus
GGTTTCATGTCATATTATAATTGATTTATTTAAAATACTTTAGAGTCTATGCAATCATAATTTTGCATTTCCAGCCTGTTAGA
AATTCGTTTTGCTGCAATACGTCGCCCAGAAAGAAACTCTGGAGAGATTTTTCTCACAAATTATTTGTCAGCTCCGCTTTTTC
ACAAAAACTGAAT

>Ycag_sat_cl24_consensus
CGATAAGTTTCTCACCCTGAACTGGATTCGATTAAAAATAAAAAATAGAAACAAAAATACAAAAAACCTCCTTCAAGAAA
ACAAAACAAAAACACTCAATTCAAGCGAAAAAATTTGCGCCGGCGATTTGAAAAGTGTATTCCGGATGGAAATCTATTGC
CAAAAAAGGTAAAGGTTGATGTAATTTAGTCAGATTTTAAGTGCTATAGGCGGCAAAAATTCGGTAATTTAGAGAAATTT
AGAAAAAAATCCTCACCTGAGGACACACACAGCCAATCTTGTTGAAATTAATGTATTGTTATCGGCAAAAAACTCGAGAA
TTAGGGAAGATTTTCACCTCGGGACACACACTGTTTACTCTCGGTGCAACACGAATAGAGTGGCGGAGCGGCGTGCGCGA
GGTAGGTCCGCTTTGTTTCGAGTTGTGGGTAGGTGCGGTCGTCTGAGGAAGGGTAAAAAAGAAAGATTTTGAAGGTATTT
TGGAATAATTTGGCACGGAACCCTGAAAAATGTCTGGAAGCGCGGCTACCAAAGGCGCAGGAGCACTCCGCGGCGAGAA
CGGCGCGATCCCGGAGGCAGGCGGTCTGCGGTTCGATTCCCGGCAATGAAAATTTGGAAAAGTGAAAATGTGAAAATTTT
TACGTAGCTGGGTCGGCTCCCGGTTTCCGACGTGCGATCCCTAACGAGGTACGACTTCACGAAAAAA

>Ycag_sat_cl27_consensus
CAGTGAGTATACCGGATTGTAGCTTTATATATATATAACTAGGGGGTGAACCCCCTGCGCGCTTACGCGCGCCAACCCCC
AGCCGCTCCGAGCTTCGCTCTCCGCGGCGCTCGGCCTAGGGCCTCGCTAGACACTTGACGTTGGGATTGTTTGAGTGGGA
ACATGTATGGTCTCGGCCTTCGGCCTCGGCCTGAACGGAGCTCGGCCTTCGGCCTCGCCGCGGCGAGGTTCCTCATCCCG
GCTTCGGCCTCGAAAAATTGTTTATTTGAAAAATTGTTCAATTTTGTTTAAAATTTTGTACTAAAAGTCGATTTTCAGTA
CAAATTCTCCATACAAAATTTTCTCATTTTCAATCGTCGATATCTCGGCCGCCATCTTGAAATGTCAAAATGTCCGATTT
TTCTCATATACCTGGGTCCTTTGACAACCCGATGCCGTTTAGGGCACGTCTCCAGCATGTGCCCAGTTGGCTGCCGACTT
TTTTGCGCAGAGGTCGATTTTTCGGGCGGCCATATTCTCGCCCCTATTTTGAATTTCGAACGTTCAAAATTTTTTTTCAC
AATTAGATCACGAAACAAACAACTATTCCAAATTTCATCCAAATCGGCCGGTAAACAAAAAAGTTGGCCCATTTGTATAG
GAGAACGCGTTCGAAATTCAAAAATCGGCTTCATAGGATGGCTGTAATTCATACATTTCCTACAACTTTCTCATACAAAA
TTTTCACGTAGCTCTTACCGTTTCCGAGGAAACCTATAGATGATCAGTCAGTCAGTCAGT

>Ycag_sat_cl40_consensus
TTGTTTTATTTTTGAATTTTCTACCATTCTGACGTGATATGCTGACACTTTTACGTGAGTTCTAGTTCTAAATTGAGATG
TTTAGTGCAGATCTGATACAGATCTATGACAAACAAAAGAGTTCTGTGTTATAAGCCTGCTAAATTTATTTAAAAATATT
A

>Ycag_sat_cl54_consensus
TAGGGTAGGTTAGGT

>Ycag_sat_cl98_consensus
CTATGTCAGCGGTGGTGTTTTATATGAGAACGGGTGAGTTTCGGAAAAGCTATGGAGAAAAAATTTTGAAAAATGAAGAA
AATTATGTGTTTTTATTTAATTACTCGAAAAATACTGAATCTAATCTTATTCTGTTTTCAGGGGCGGATAGCTGGGAGTG
AGACGCTTACAAAAAGGTGCGGCACGAGCAGGTAAGTGAAATTTTCAGAATTAGTTTTTCGGTGATGTATGAAACTATTC
TATCACTAAGGTGAACTTTTTGCTTGACGCTTAGTATGGAGTTCGACGAAATACCACCCTATCACCATAAAAGTGGTGTC
TATCGACGCGGGGGTGTCCCG

>Ycag_sat_cl214_consensus
AGTAAAAATTCCTTTTGAAAAGGTGGTATTTTCCTTCCTCAATTACCATCCGATTCGGAAAACTGCAAAATTCCTACCAA
ATTTGGAAGGTTATTTATACTATCCGTCTTATTCGGAAGAAAATATCGAAAGGAAAATATTCAGAAGGATTATACTAAGA
AGGAAACACCTTCCAGCGCAATTTTCCTACTAAAAAAGGAAGGAAATACTTTTCCTTCCTAATTTTTACCTTTCAAAACA
GCAGGAAATTTTTTGT

Table S4: Analysis of repetitive DNA by RepeatMasker. Species specific repeat library was constructed de novo with RepeatModeler and used for repeat prediction. In total, 292,801,135 bp (46.75 %) of the assembly was annotated as repetitive elements.

Class	Number of elements	Length occupied (bp)	Percentage of sequence
SINEs	209,916	31,673,948	5.06
ALUs	0	0	0.00
MIRs	0	0	0.00
LINEs	368,058	66,955,288	10.69
LINE1	0	0	0.00
LINE2	103,670	21,198,685	3.38
L3/CR1	5,764	1,914,424	0.31
LTR elements	15,639	11,265,711	1.80
ERVL	0	0	0.00
ERVL-MaLRs	0	0	0.00
ERV_class l	0	0	0.00
DNA elements	94,509	20,825,156	3.33
hAT-Charlie	4,032	758,464	0.12
TcMar-Tigger	0	0	0.00
Unclassified interspersed repeats	897,499	152,283,337	24.32
Small RNA	55,539	8,781,860	1.40
Satellites	12,250	3,584,491	0.57
Simple repeats	117,680	6,423,689	1.03
Low complexity	12,352	589,128	0.09

Table S5. Estimates of evolutionary divergence for a selected group of genes

Shown is the number of base differences per site (p-spacing) between sequences. Standard error estimates are indicated above the diagonal. All ambiguous positions were removed for each pair of sequences (pairwise deletion option). Evolutionary analyses were performed in MEGA X [1].

Tibi – *Tioneola bisselliela* (Tineidae), Ypca –*Yponomeuta cagnagella*, Ypev - *Yponomeuta evonymella*.

Metabolic genes

IDGF	Tibi	Ypca	Ypev
Tibi		0.0125	0.0125
Ypca	0.3102		0.0025
Ypev	0.3063	0.0084	

β-gal	Tibi	Ypca	Ypev
Tibi		0.0112	0.0112
Ypca	0.4102		0.0028
Ypev	0.4129	0.0149	

G6PD	Tibi	Ypca	Ypev
Tibi		0.0111	0.0111
Ypca	0.2468		0.0010
Ypev	0.2468	0.0014	

TPI	Tibi	Ypca	Ypev
Tibi		0.0166	0.0166
Ypca	0.2930		0.0019
Ypev	0.2930	0.0027	

Silk genes

Fhx/P25	Tibi	Ypca	Ypev
Tibi		0.0192	0.0193
Ypca	0.4568		0.0030
Ypev	0.4599	0.0061	

FibL	Tibi	Ypca	Ypev
Tibi		0.0185	0.0182
Ypca	0.4716		0.0051
Ypev	0.4859	0.0194	

FibH5'	Tibi	Ypca	Ypev
Tibi		0.0159	0.0160
Ypca	0.4335		0.0016
Ypev	0.4357	0.0032	

FibH3'	Tibi	Ypca	Ypev
Tibi		0.0150	0.0150
Ypca	0.4752		0.0039
Ypev	0.4787	0.0221	

1. Kumar S., Stecher G., Li M., Knyaz C., and Tamura K. (2018). MEGA X: Molecular Evolutionary Genetics Analysis across computing platforms. Molecular Biology and Evolution 35:1547-1549.

Table S6 – Data for Figure 4 (qPCR)

	FibL	Ssp1	Zon1	Zon2	Zon3	Zon4	FB1L	SLP	PXD	VAL	SPL	ACHP	TRPL	FB2L	BNB	IDGF	AGRL
SilkG1	25,63632	1,473971	0,002088	0,010001	0,350301	0,056009	4,958831	0,051356	0,001919	0,009386	0,005654	0,002899	0,008125	0,008953	0,037589	0,026355	0,014519
SilkG2	30,02627	1,847717	0,002427	0,008352	0,153184	0,055052	3,167475	0,034276	0,00149	0,010857	0,006995	0,00344	0,008529	0,008995	0,03305	0,028544	0,015646
SilkG3	31,39293	2,20749	0,002743	0,009017	0,180491	0,056391	3,689265	0,041714	0,001878	0,012656	0,009056	0,004158	0,009574	0,011438	0,046644	0,033054	0,018379
Gut1	0,004308	0,000582	0,000235	0,000614	0,000215	0,000118	0,001372	2,6E-05	2,16E-05	0,000557	0	0,035567	0,139984	0,000298	0,034511	0,099449	0,198197
Gut2	0,005662	0,000565	0,000297	0,000811	9,56E-05	0,000106	0,000948	1,21E-05	1,35E-05	0,000809	5,3E-05	0,046391	0,139017	0,000248	0,036614	0,113413	0,222803
Gut3	0,006556	0,000672	0,000306	0,000631	9,23E-05	7,42E-05	0,001127	1,84E-05	1,12E-05	0,000835	5,05E-05	0,055939	0,14161	0,000346	0,042052	0,118719	0,275555
FatB1	0,213533	0,019313	2,6E-05	0,001905	0,001242	0,000192	0,006864	0,000116	3,51E-05	0,000918	0	1,057018	0,002003	0,023249	0,021723	1,751774	0,483982
FatB2	0,267364	0,024018	6,1E-05	0,001293	0,000506	0,000184	0,004765	4,25E-05	4,01E-05	0,000813	9,11E-05	1,217004	0,001744	0,01762	0,02477	1,79046	0,45809
FatB3	0,306191	0,030496	8E-05	0,001071	0,000515	0,000273	0,005499	6,36E-05	7,73E-05	0,000868	4,53E-06	1,313425	0,002113	0,020953	0,026277	1,949436	0,517725
Integ1	0,001511	0,000453	0,000369	0,001213	0,003173	0,001017	0,000398	0	0,000225	0,000458	0,000494	0,550953	0,000283	0,005563	0,073386	1,5814	0,159553
Integ2	0,002104	0,000661	0,000415	0,001695	0,001665	0,001183	0,00035	2,46E-05	0,000257	0,000681	0,00036	0,686184	0,000319	0,005576	0,06688	1,812539	0,204786
Integ3	0,002038	0,000847	0,000536	0,001496	0,001681	0,001159	0,000397	1,77E-05	0,00024	0,000722	0,000456	0,793701	0,000373	0,006201	0,079348	2,191505	0,242703
