# Supplementary material for: Genome sequence and silkomics of the spindle ermine moth, Yponomeuta cagnagella, representing the early diverging lineage of the ditrysian Lepidoptera
Source: Commun Biol. 2022 Nov 23;5:1281. doi: 10.1038/s42003-022-04240-9 (PMC9684489; doi:10.1038/s42003-022-04240-9)
Supplement: Supplementary file 3 — Reporting Summary [file 42003_2022_4240_MOESM3_ESM.pdf]

nature portfolio

Corresponding author(s): Michal Zurovec COMMSBIO-22-0323-A

Last updated by author(s): Nov/5/22

Reporting Summary

Nature Portfolio wishes to improve the reproducibility of the work that we publish. This form provides structure for consistency and transparency in reporting. For further information on Nature Portfolio policies, see our Editorial Policies and the Editorial Policy Checklist.

Statistics

For all statistical analyses, confirm that the following items are present in the figure legend, table legend, main text, or Methods section.

n/a	Confirmed	
☐	☒	The exact sample size (*n*) for each experimental group/condition, given as a discrete number and unit of measurement
☐	☒	A statement on whether measurements were taken from distinct samples or whether the same sample was measured repeatedly
☐	☒	The statistical test(s) used AND whether they are one- or two-sided<br>*Only common tests should be described solely by name; describe more complex techniques in the Methods section.*
☒	☐	A description of all covariates tested
☒	☐	A description of any assumptions or corrections, such as tests of normality and adjustment for multiple comparisons
☒	☐	A full description of the statistical parameters including central tendency (e.g. means) or other basic estimates (e.g. regression coefficient) AND variation (e.g. standard deviation) or associated estimates of uncertainty (e.g. confidence intervals)
☒	☐	For null hypothesis testing, the test statistic (e.g. *F*, *t*, *r*) with confidence intervals, effect sizes, degrees of freedom and *P* value noted<br>*Give P values as exact values whenever suitable.*
☒	☐	For Bayesian analysis, information on the choice of priors and Markov chain Monte Carlo settings
☒	☐	For hierarchical and complex designs, identification of the appropriate level for tests and full reporting of outcomes
☒	☐	Estimates of effect sizes (e.g. Cohen's *d*, Pearson's *r*), indicating how they were calculated

Our web collection on statistics for biologists contains articles on many of the points above.

Software and code

Policy information about availability of computer code

Data collection	Oxford Nanopore reads were sequenced on the Nanopore PromethION platform by Novogene (HK) Co, Ltd. (Hong Kong, China); The cDNA library was sequenced on Illumina platform 2×150 bp (paired end reads) with MiSeq.
Data analysis	Trimmomatic (version 0.36); NanoFilt (version 2.7.1); FM-index Long Read Corrector (FMLRC version 1.0.0; Flye (version 2.8), purge_dups pipeline (version 1.0.1); POLCA (MaSuRCA version 3.4.2); QUAST (version 4.6.3); BUSCO tool suite (version 5.2.2); Kraken 2 (version 1.0); RepeatModeler (version 1.0); RepeatMasker (version 4.0); rnaSPAdes assembler (version 3.13.1); Salmon (version 1.0.0); DIAMOND protein aligner (version 0.9.27.128); Transdecoder (version 5.5); Lasergene PrimerSelect (DNASTAR, Madison, USA); MaxQuant (version 1.5.3.8);

For manuscripts utilizing custom algorithms or software that are central to the research but not yet described in published literature, software must be made available to editors and reviewers. We strongly encourage code deposition in a community repository (e.g. GitHub). See the Nature Portfolio guidelines for submitting code & software for further information.

Data

Policy information about availability of data

All manuscripts must include a data availability statement. This statement should provide the following information, where applicable:

- Accession codes, unique identifiers, or web links for publicly available datasets
- A description of any restrictions on data availability
- For clinical datasets or third party data, please ensure that the statement adheres to our policy

Provide your data availability statement here.

Human research participants

Policy information about studies involving human research participants and Sex and Gender in Research.

Reporting on sex and gender	*Use the terms sex (biological attribute) and gender (shaped by social and cultural circumstances) carefully in order to avoid confusing both terms. Indicate if findings apply to only one sex or gender; describe whether sex and gender were considered in study design whether sex and/or gender was determined based on self-reporting or assigned and methods used. Provide in the source data disaggregated sex and gender data where this information has been collected, and consent has been obtained for sharing of individual-level data; provide overall numbers in this Reporting Summary. Please state if this information has not been collected. Report sex- and gender-based analyses where performed, justify reasons for lack of sex- and gender-based analysis.*
Population characteristics	*Describe the covariate-relevant population characteristics of the human research participants (e.g. age, genotypic information, past and current diagnosis and treatment categories). If you filled out the behavioural & social sciences study design questions and have nothing to add here, write "See above."*
Recruitment	*Describe how participants were recruited. Outline any potential self-selection bias or other biases that may be present and how these are likely to impact results.*
Ethics oversight	*Identify the organization(s) that approved the study protocol.*

Note that full information on the approval of the study protocol must also be provided in the manuscript.

Field-specific reporting

Please select the one below that is the best fit for your research. If you are not sure, read the appropriate sections before making your selection.

☒ Life sciences ☐ Behavioural & social sciences ☐ Ecological, evolutionary & environmental sciences

For a reference copy of the document with all sections, see nature.com/documents/nr-reporting-summary-flat.pdf

Life sciences study design

All studies must disclose on these points even when the disclosure is negative.

Sample size	The number of individuals used is specified for each method.
Data exclusions	No data were excluded.
Replication	Each sample was analyzed in three replicates and the results were reproducible.
Randomization	Samples were prepared from animals randomly collected in nature.
Blinding	Samples were blinded during data collection and analysis.

Reporting for specific materials, systems and methods

We require information from authors about some types of materials, experimental systems and methods used in many studies. Here, indicate whether each material, system or method listed is relevant to your study. If you are not sure if a list item applies to your research, read the appropriate section before selecting a response.

Materials & experimental systems

n/a	Involved in the study
☒	☐ Antibodies
☒	☐ Eukaryotic cell lines
☒	☐ Palaeontology and archaeology
☐	☒ Animals and other organisms
☒	☐ Clinical data
☒	☐ Dual use research of concern

Methods

n/a	Involved in the study
☒	☐ ChIP-seq
☐	☒ Flow cytometry
☒	☐ MRI-based neuroimaging

Animals and other research organisms

Policy information about studies involving animals; ARRIVE guidelines recommended for reporting animal research, and Sex and Gender in Research

Laboratory animals

For laboratory animals, report species, strain and age OR state that the study did not involve laboratory animals.

Wild animals

For flow cytometry, we used Yponomeuta cagnagella (Hübner, 1813) males from laboratory mass rearing started with larvae collected in Levin (Czech Republic). For sequencing and proteomic analyses, Y. cagnagella egg batches were collected in Watergraafsmeer (Amsterdam, The Netherlands). Hatched larvae were reared on twigs of their food plant, Euonymus europaeus (Linnaeus, 1753), until pupation. Pupae were sexed by their morphology, frozen in liquid nitrogen and stored for DNA extraction at -80°C. Yponomeuta evonymella (Linnaeus, 1758) larvae were collected in Amsterdam (The Netherlands) and Vrabce (Czech Republic). Adults of the Mediterranean flour moth Ephestia kuehniella (Zeller, 1879; Lepidoptera, Pyralidae) were obtained from laboratory wild-type strain WT-C 29. Larvae of the wax moth Galleria mellonella (Linnaeus, 1758; Lepidoptera, Pyralidae) were from laboratory strain that was originally established from specimens found in Ceske Budejovice (Czech Republic).

Reporting on sex

Indicate if findings apply to only one sex; describe whether sex was considered in study design, methods used for assigning sex. Provide data disaggregated for sex where this information has been collected in the source data as appropriate; provide overall numbers in this Reporting Summary. Please state if this information has not been collected. Report sex-based analyses where performed, justify reasons for lack of sex-based analysis.

Field-collected samples

Animals were kept at room temperature (long day) or used directly after collection.

Ethics oversight

n/a insects

Note that full information on the approval of the study protocol must also be provided in the manuscript.

Flow Cytometry

Plots

Confirm that:

☒ The axis labels state the marker and fluorochrome used (e.g. CD4-FITC).

☒ The axis scales are clearly visible. Include numbers along axes only for bottom left plot of group (a 'group' is an analysis of identical markers).

☒ All plots are contour plots with outliers or pseudocolor plots.

☒ A numerical value for number of cells or percentage (with statistics) is provided.

Methodology

Sample preparation

Flow cytometry was used to estimate the genome size of Y. cagnagella. The methodology differs from flow cytometry applications in immunological and related research, and some of the usual parameters are not relevant to genome size measurement. In particular, only two populations are usually of interest (2C nuclei of the sample and the standard), and the focal variable is the mean fluorescence of each of them (to calculate the ratio of mean fluorescence); fluorescence must be measured accurately. One figure is a histogram (one parameter only), the figure illustrating gating strategy prior the histogram analysis is a pseudocolour plot (see below). A numerical value for number of cells or percentage is not relevant for the genome size samples (the number/percentage of cells of each population is not the focal variable).

Instrument

Partec CyFlow SL flow cytometer (Partec, Münster, Germany; now Sysmex) equipped with a 100 mW 532 nm (green) solid state laser.

Software

Data were analyzed using FlowJo 10 software (TreeStar, Inc., Ashland, OR, USA).

Cell population abundance

As explained above, cell (nuclei) population abundance is not the focal variable and is not relevant for genome size

Cell population abundance	measurements. The recommended minimum of 1,000 nuclei for each peak (i.e., 2C peaks of the sample and the standard) is reached in all samples.
Gating strategy	The figure has been added (Fig. S2B).

☒ Tick this box to confirm that a figure exemplifying the gating strategy is provided in the Supplementary Information.
